# Thymol as Starting Material for the Development of a Biobased Material with Enhanced Antimicrobial Activity: Synthesis, Characterization, and Potential Application

**DOI:** 10.3390/molecules29051010

**Published:** 2024-02-26

**Authors:** Giovana A. Parolin, Vitor G. Vital, Suzan P. de Vasconcellos, João Henrique G. Lago, Laura O. Péres

**Affiliations:** 1 Laboratory of Hybrid Materials, Chemistry Department, Federal University of São Paulo, Diadema 09913-030, SP, Brazil; giovana_parolin@hotmail.com; 2 Multidisciplinary Laboratory of Health and Environment Sciences, Department of Pharmaceutical Sciences, Federal University of São Paulo, Diadema 09913-030, SP, Brazil; vitor.vital@unifesp.br (V.G.V.); suzan.pantaroto@unifesp.br (S.P.d.V.); 3 Laboratory of Chemical Biology, Center for Natural and Human Sciences, Federal University of ABC, Santo Andre 09210-580, SP, Brazil; joao.lago@ufabc.edu.br

**Keywords:** thymol, polymerization, polythymol, biofilm dispersion, antimicrobial activity

## Abstract

A biobased material, polythymol (PTF), was prepared using thymol, a monoterpene obtained from the essential oil of *Thymus vulgaris* (Lamiaceae), as a starting material with the aim of enhancing the antimicrobial properties of this natural product. Initially, different processes were performed in order to optimize the reaction conditions to obtain a macromolecule with a high purity and yield. PTF was characterized using different techniques, such as NMR, infrared, UV-Vis, and thermogravimetric analyses. The antimicrobial activity of both PTF and thymol was evaluated against different microorganisms, including *S. aureus*, *E. coli*, *P. aeruginosa*, and *C. albicans*. The obtained MIC values showed a higher potential for PTF than the monomer thymol—for example, against *S. aureus* (500 and 31.5 µg·mL^−1^ for thymol and PTF, respectively). Therefore, the obtained results show that the polymerization of thymol afforded more active biomaterial than the starting monomeric antimicrobial compound (thymol), suggesting that PTF is an important biomaterial.

## 1. Introduction

Thymol is an aromatic monoterpene occurring in essential oils from different plant species, especially those from the genera *Thymus* and *Ocimum* (Lamiaceae) [1]. This compound occurs as the main constituent of the essential oils of these plants, which are used in the food industry due to their flavoring and preservative properties [2]. Thymol can also be used for biological applications due to its strong bactericidal activity [1]. Recently, some studies have reported on several applications of thymol (isolated or combined in composites), such as food packaging [3], agricultural products [4], industrial [5] and pharmacokinetic areas [6], and drug delivery with encapsulation [7], and the studies aimed to overcome its hydrophobicity, volatility, and other limitations that could narrow its application. Its antimicrobial activity is directly related to the functional groups present in the molecule, especially the hydroxyl group at the aromatic ring [8]. The mechanism of action is mainly based on damage and dysfunction in the plasma membrane of microorganisms (MOs). It is highlighted that thymol can cause the rupture of the biofilm produced by microorganisms, which can prevent the penetration of antibiotics and disinfectants into the cell. This ability to form biofilms is attached to the resistance of bacteria and fungi to many developed products [9]. Considering the occurrence of this compound in natural sources, our group decided to use thymol as a starting material to prepare a new polymeric material, named polythymol (PTF).

It is well known that polymers are interesting due to their capacity to form films, in addition to other properties that improve with an increase in the oligomeric chain, such as mechanical and thermal resistance [10]. This fact further expands the possible applications for these materials. Moreover, it can make them useful in pharmacological aspects beyond traditional plastics and rubber applications, especially due to their biocompatibility, biodegradability, and good mechanical properties [11,12,13,14,15,16]. Therefore, there has been an increase in studies on biobased polymers as a topic to expand the research in this area [17,18]. Biobased materials and derivates can be used as monomers, for example, to change small molecules into macromolecules, which can improve their properties, allowing the use of the synthesized biobased product in interdisciplinary fields [19]. In this sense, the polymerization of thymol can provide a more effective compound in comparison to its natural monomeric compound, especially against microorganisms. Additionally, one of the advantages of using these materials is that they can be added in significantly smaller quantities if utilized with a matrix, and, in the final product, the properties will be the same or better as those of the isolated components.

In previous works, it has been shown that this macromolecular material presents bioactivity that can be used to treat cutaneous leishmaniasis [20] and Chagas disease [21]. Based on these aspects, this work aimed the synthesize a polymer derived from thymol with improved properties of the macromolecule in relation to those of the monomer against bacterial species, such as *Staphylococcus aureus*, *Escherichia coli*, and *Pseudomonas aeruginosa*, aiming for future bioapplications if these molecules can show bioactivity even higher than that of thymol.

## 2. Results and Discussion

To obtain PTF in higher yields, different reactions were performed by varying some parameters, such as the duration of the reaction, temperature, and work-up. The reactions of Method 1 were conducted using acid catalysis, which provided yields from 28 to 83% with molar masses ranging from 650 to 1295 g·mol^−1^ (Table 1). Entry 1 provided a material with a relatively higher molar mass (1295 g·mol^−1^), but with a reduced yield. Otherwise, the yields of Entries 2–4 were higher, but the isolated polymers displayed a reduced molecular mass (650 from 793 g·mol^−1^).

In order to compare the effectiveness of the reaction using a basic medium, the reaction was conducted using Method 2: 40% NaOH was used as the catalyst with different temperatures (60 and 80 °C) and while varying the duration of the reaction (24 and 48 h) to obtain a material with a high molecular mass combined with satisfactory yields. Entry 5 provided a material with 488 g·mol^−1^, while Entry 6 provided a higher molar mass (1375 g·mol^−1^). Entries 7–10 exhibited lower yields (19 to 49%) and molar masses (903, 1106, 373, and 715 g·mol^−1^, respectively). In the GPC curves, a distribution of the molar mass of the oligomers was observed along the elution of the materials, forming almost Gaussian curves. For all the synthesized products, it was possible to observe broad curves, which indicated a polydispersed material (in Table 1, the polydispersity index (PDI) is between 1.38 and 1.99), as well as the presence of peaks in the curves, which implies that there were groups with distinct molar masses. These values of the PDI above 2.0 are fitting for this kind of material [22], which normally has a polydispersity between 1.7 and 3.0 [23].

It is important to mention that the performed reactions were monitored using NMR. In many cases, it was possible to observe the presence of three peaks assigned to the hydrogens of the aromatic ring of thymol (H-5, H-2, and H-1) at δ 6.57–7.10 [24]. Only for Entries 6, 7, 8, and 10, the reaction effectively provided pure PTF without the presence of subproducts, with yields of 62, 44, 49, and 34%, respectively (NMR data—Figures S5 and S6, in Supplementary Materials). Thus, in these cases, the obtained results indicated that the reaction conducted using basic catalysis at 60 °C for 2 h with an equimolar proportion for thymol and formaldehyde and neutralization with HCl afforded, comparatively, the best yield combined with purity as well as a higher molar mass for the PTF (Entry 6). Additionally, the PTF obtained in Entry 6 was washed and extracted successively with CHCl_3_ and CH_3_OH in order to obtain two fractions: PTF/CHCl_3_ and PTF/CH_3_OH (the fractions were named according to the solvent utilized in the extraction process), which displayed similar NMR and FTIR profiles.

**Figure 1:**
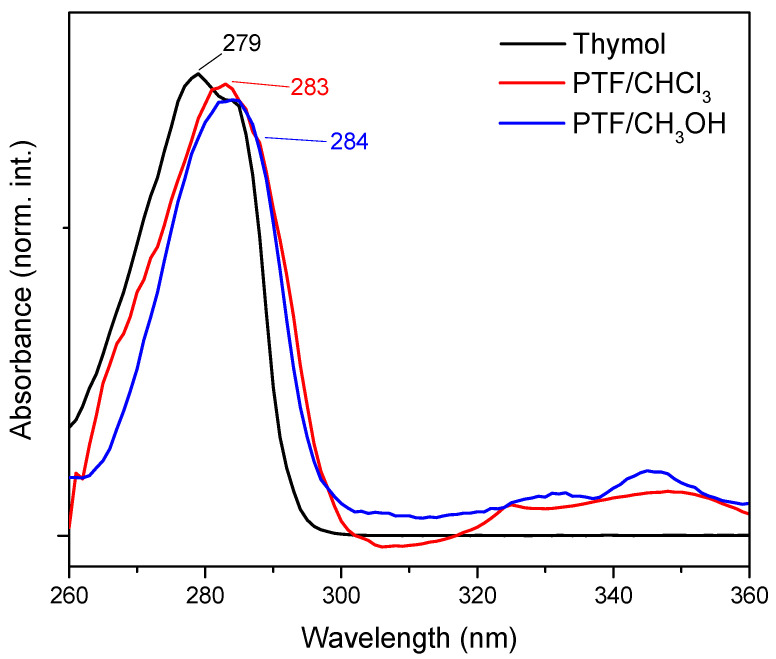
UV-Vis absorption spectrum of thymol (black line), PTF/CHCl_3_ (red line), and PTF/CH_3_OH (blue line) with DMSO as the solvent.

The results of the UV-Vis analysis (Figure 1) indicated a displacement of the maximum absorption band for thymol (λ_max_ = 279 nm) in comparison to oligomers. For PTF/CHCl_3_, the maximum absorption band was located at 283 nm, and for PTF/CH_3_OH, it was located at 284 nm and was assigned to the aromatic groups present in all samples, which are referred to as electronic transitions, π → π*. In this way, the small bathochromic displacement observed suggests that, with an increase in aromatic rings in the same molecule, the intra- and intermolecular interactions are promoted, which could cause a decrease in the E_gap_ and, consequently, an increase in the maximum absorption for materials with a higher molar mass than thymol, for example [20]. However, it was not possible to perceive any significant alteration between the oligomers that could differentiate them through optical characterization techniques such as UV-Vis absorption. The same fact was observed for the FTIR analysis (Figure S4 in Supplementary Materials), once the infrared spectra for fractions were similar, since the exact same chemical bonds were observed for the products (also confirmed by NMR data) (what differentiates these materials from each other is the chain size, the polydispersity, and the yield of the reactions).

A thermogravimetric analysis was conducted for the thymol, PTF/CHCl_3_, and PTF/CH_3_OH obtained from Entry 6 (Table 2). The obtained results indicated only one event of thermal degradation for thymol with 100% mass loss, which is consistent with what is expected for this material [25]. For the PTF/CHCl_3_ and PTF/CH_3_OH fractions, similar curves were observed with a small variation in the slope of the lines (Figure S7 in Supplementary Materials). For example, for the PTF/CHCl_3_ sample, two main events were observed: the first one was from 88 to 260 °C with 17% mass loss, attributed to dehydration and the initiation of the thermal degradation of the oligomer through random chain scission, and this was followed by the second event, from 260 to 530 °C with 76% mass loss, attributed to complete chain degradation. In the case of oligomer PTF/CH_3_OH, a slighter drop was observed with the formation of small degrees, probably due to the degradation of the material, occurring firstly through the elimination of side groups, followed by the depolymerization process.

For the GPC analysis, in this case, for each fraction of Entry 6, a difference was observed in the size of the two samples: for PTF/CHCl_3_, the weighted average molar mass was found to be 1182.0 g·mol^−1^, while for PTF/CH_3_OH, the value was 1375.0 g·mol^−1^, which was in agreement with the polydispersity data observed in the primary GPC results for Entry 6. Despite the differences between these values, it is possible to infer that, with an increase in the chain size, some properties of the oligomers may differ, such as the solubility in different solvents (for example, chloroform or methanol) or the biological properties, as opposed to the thermal and optical properties, which, in this specific case, were not significantly affected.

The antimicrobial effects of both oligomers against *Staphylococcus aureus*, *Escherichia coli*, *Pseudomonas aeruginosa*, and *Candida albicans* were determined in vitro and the obtained results are presented in Table 3. It was possible to observe that thymol displayed bactericidal activity with a concentration of 500 μg/mL against all the tested bacteria. Similarly, for *E. coli*, the samples PTF/CHCl_3_ and PTF/CH_3_OH showed the same bioactivity. For *P. aeruginosa*, PTF/CH_3_OH displayed a similar MIC to that determined for thymol (500 μg·mL^−1^), but not for PTF/CHCl_3_ (which presented no activity, in this specific case). For the sample PTF/CH_3_OH, the bioactivity against the fungus *C. albicans* was shown with an MIC of 500 μg·mL^−1^, which was not observed in the widely used monomer for bioapplication purposes. 

The values for *S. aureus* are highlighted, where the MIC significantly decreased compared to that of thymol, from 500 μg·mL^−1^ to 31.2 and 62.5 μg·mL^−1^ for the samples with lower and higher molar masses, respectively. As previously mentioned, since the mechanism of action for the monomer thymol promotes damage and dysfunction in the plasma membrane of microorganisms through the presence of functional groups (hydroxyl, in this case) and delocalized electrons, it was inferred that the higher the amount of hydroxyl and delocalized electrons (as in the case of both oligomers), the more the cell damage increases and, consequently, the lower the MIC is of the samples against different pathogens, which is a great advantage [8].

Thymol is a compound with well-known biocidal properties that has been widely studied in research. A study conducted by García-Salinas [26] evaluated the activity of thymol against various bacterial species, including *Staphylococcus aureus*, *Escherichia coli*, and *Pseudomonas aeruginosa*. The results showed that thymol exhibited significant activity against these organisms, decreasing cell viability by up to 99%. Other analyses were conducted with thymol linked to other compounds to evaluate its antimicrobial activity against the same microorganisms. These studies demonstrated that the activity of thymol can be further enhanced when combined with other compounds, such as polythymol [27].

**Figure 2:**
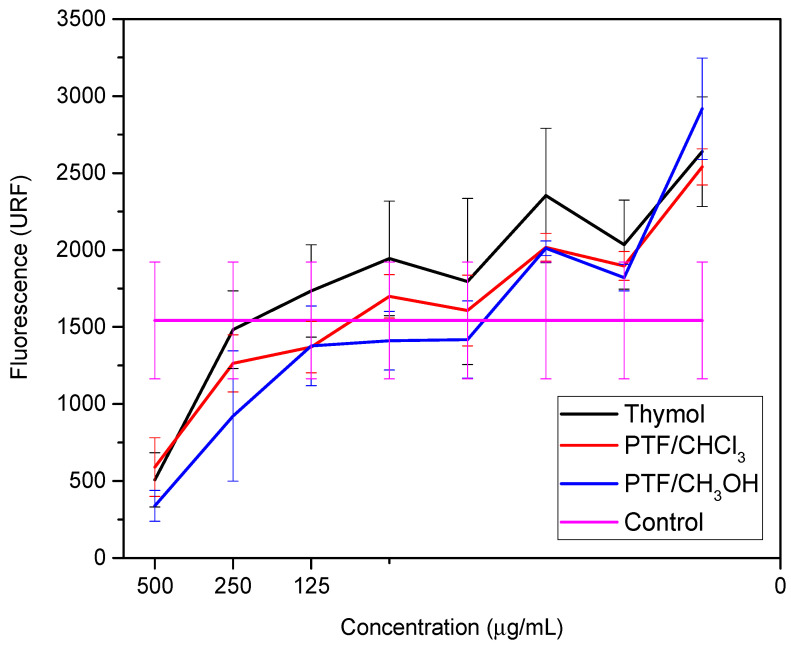
Cell viability at different concentrations of thymol, PTF/CHCl_3_, and PTF/CH_3_OH from the metabolization of resazurin to resofurin.

**Figure 3:**
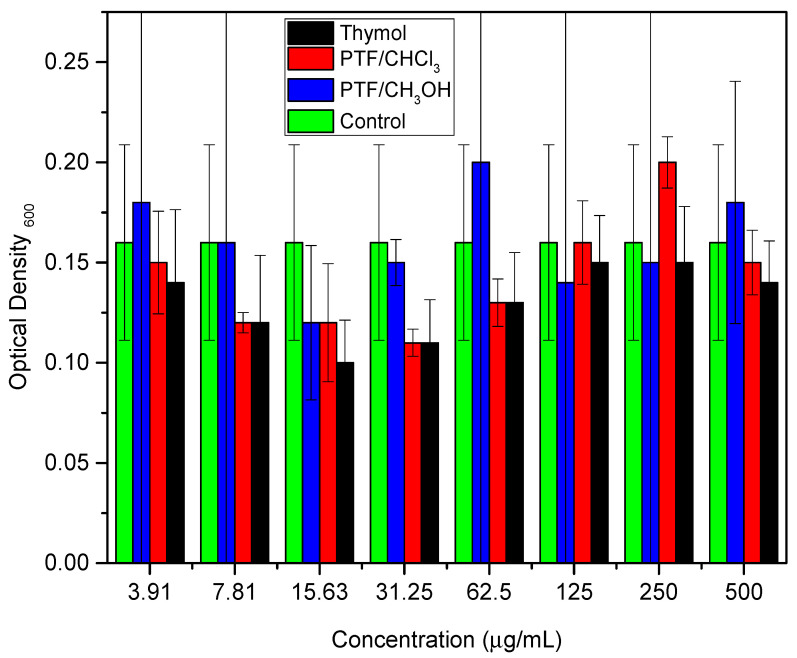
Colorimetric quantification of biomass, comparing different concentrations of thymol, PTF/CHCl_3_, and PTF/CH_3_OH.

These studies emphasized the relevance of thymol as a promising option for the development of new materials with antimicrobial activity against bacteria, including *E. coli*. The combination of thymol with other biocidal compounds can result in synergy, further improving its biocidal activity. Similarly, the biofilm dispersal assay was conducted for thymol and both oligomer fractions to compare these two samples with their monomer. Two bioassays were performed to evaluate the samples against the biofilm-producing microorganism: a cell viability assay (Figure 2) and a biomass quantification assay (Figure 3) (Tables S1 and S2, respectively, in Supplementary Materials).

*Pseudomonas aeruginosa* is described as an opportunistic pathogen that can cause infections in humans, particularly those with compromised immune systems [28]. *P. aeruginosa* infections are difficult to treat due to the bacteria’s inherent resistance to many antibiotics, as well as its ability to form biofilms that protect it from the immune system and antibiotics [29]. In recent years, there has been increasing interest in the use of natural compounds as alternative therapies to combat *P. aeruginosa* infections, such as thymol [30]. Here, we can observe in Figure 3 that thymol exhibits biofilm dispersion activity at a concentration of 15.63 µg·mL^−1^, the same value observed for polymyxins. Other studies have shown that thymol possesses antimicrobial properties, including against *P. aeruginosa*, and has been proposed as a potential treatment option for *P. aeruginosa* infections [31]. In a study by Memar et al. [32], it was found that thymol significantly reduces the expression of genes involved in biofilm formation and virulence in *P. aeruginosa*. Additionally, thymol was able to disperse pre-formed biofilms of *P. aeruginosa*, which may have important implications for the treatment of *P. aeruginosa* infections.

Therefore, the synthesized macromolecules can be applied in several functions [23] that require bactericidal activity and, in the case of the higher-molar-mass material, antifungal activity, to improve the bioactivity as well as to reduce the amount of needed material, for example. As previously reported [20], polythymol displayed activity against promastigote forms of *Leishmania (Leishmania) amazonensis* with an EC_50_ of 8.4 ± 0.8 μg·mL^−1^ and a reduced toxicity to macrophages (CC_50_ > 100 μg·mL^−1^ and SI > 11.9). Together, these results suggest that polythymol can be considered a new biobased drug for treating different diseases, including cutaneous leishmaniasis as well as those caused by microorganisms.

## 3. Materials and Methods

### 3.1. General Experimental Procedures

The solvents, reactants, and materials were purchased from Synth (Diadema, SP, Brazil) and Sigma-Aldrich Co. (St. Louis, MO, USA). LREIMS spectra were obtained using a Shimadzu chromatograph model GCMS-QP2010 equipped with a flame ionization detector (FID), using an RtX-5 capillary column (5% phenyl, 95% polydimethylsiloxane, and a 30 m × 0.25 mm × 0.25 μm film thickness) and helium as the carrier gas (1 mL·min^−1^), and interfaced with an MS-QP-2010 and a quadrupole mass analyzer with impact electron ionization, operating at 70 eV. ^1^H and ^13^C NMR spectra were recorded on a Bruker DPX-300 spectrometer, operating at 300 MHz for ^1^H and 75 MHz for ^13^C nuclei. CDCl_3_ or CD_3_OD (Aldrich) were used as the solvent and internal standard. Chemical shifts were reported in δ units (ppm) and coupling constants (*J*) were reported in Hz. FTIR spectra were obtained using a Shimadzu infrared spectrometer model IR Prestige-21 using a diffusion reflectance accessory—from 4000 to 400 cm^−1^, with 4 cm^−1^ of resolution and 1024 scans. Gel permeation chromatography (GPC) experiments were conducted on an Agilent 1100 with a refractive index detector using THF as the solvent at 1 mL·min^−1^ at 23 °C. The used columns (1PLgel-mixed C and 1PLgel-mixed B) were calibrated with polystyrene (PS) standards. UV-Vis spectra were recorded using a Thermo Scientific Evolution 201 with a quartz cuvette, with 1 cm of an optical path and dimethyl sulfoxide (DMSO) as the solvent. A thermogravimetric analysis was performed using SDT-650 equipment (TA Instruments) ranging from 20 to 800 °C at 10 °C·min^−1^, and with an inert nitrogen atmosphere at 50 mL·min^−1^.

### 3.2. Isolation of Thymol

Thymol was isolated from essential oil extracted from fresh leaves of *Thymus vulgaris* according to previous work [20,21] and characterized using LREIMS, ^1^H, and ^13^C NMR as well as FTIR spectroscopy (Figures S1–S4, respectively, in Supplementary Materials).

Thymol. Pale yellow oil. ¹H NMR (300 MHz, CDCl_3_) δ_H_ 1.24 (d, *J* = 6.9 Hz, H-9 and H-10), 2.26 (s, H-7), 3.16 (m, H-8), 4.72 (s, H-11), 6.57 (s, H-5), 6.72 (d, *J* = 7.8 Hz, H-2), and 7.07 (d, *J* = 7.8 Hz, H-1). ¹³C NMR (75 MHz, CDCl_3_) δ_C_ 20.8 (C-7), 22.7 (C-9 and C-10), 26.7 (C-8), 116.0 (C-5), 121.7 (C-1), 126.2 (C-2), 131.4 (C-3), 136.6 (C-6), and 152.6 (C-4). IR ν/cm^−1^: 4000–3290 (ν_O-H_ and ν_CH_ arom), 2994–2820 (ν_C-H_ aliph), 1875 (δ_CH_), 1620 (ν_C=C_ arom), 1090 (ν_C-O_), and 950–730 (δ_CH_). LREIMS (70 eV) *m*/*z* (int. rel): 150 (35), 135 (100), 115 (16), 107 (11), 91 (16), 77 (5), and 65 (4).

### 3.3. Synthesis of Poly(thymolformaldehyde) (PTF)

**Figure 4:**
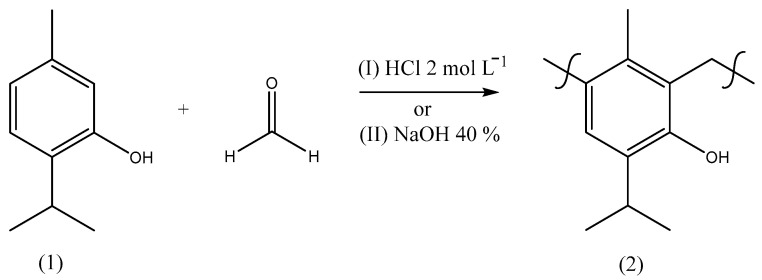
Synthetic route scheme to prepare poly(thymolformaldehyde) through methods I or II using different conditions (Table 1).

After the isolation of thymol, different procedures for the polymerization [20] of this monoterpene with formaldehyde were carried out based on the literature with adaptations [33,34] to provide a material with the chemical structure indicated by Figure 4. In order to prepare a material with polymeric characteristics, these reactions were optimized by trials to obtain a product with a high molar mass combined with the best purity and yield (Table 4), using different reaction times, temperatures, catalysts, treatments, and ratios of reactants as well as the use of a microwave (MW).

#### 3.3.1. Method I—Acid Catalyst

The reaction was carried out using an equimolar amount of thymol and formaldehyde (3.30 mmol) for 4 h at 60 °C with 1 mL of HCl (2 mol·L^−1^), as indicated in Entry 1, Table 4. The material was neutralized with NaOH (1 mol·L^−1^), filtered, washed with H_2_O, and dried under a vacuum to afford a yellow material. Additionally, the reaction was performed using a microwave (MW) conducted at 300 W for 1 or 3 h at 80 °C (Entries 2 and 3, Table 4), with the same amounts of reactants and catalyst in a microwave test tube. The product was neutralized with NaHCO_3_ (1 mol·L^−1^), extracted with CHCl_3_, dried over MgSO_4_, and filtered. After the evaporation of the solvent under reduced pressure, a viscous yellow solid was obtained. The reaction was also performed by varying the ratio of thymol and formaldehyde to 1:4, respectively (Entry 4, Table 4). The crude material was chromatographed over a Sephadex LH-20 column (4 × 50 cm) using CHCl_3_:MeOH 1:1 as the mobile phase to afford pure poly(thymolformaldehyde).

#### 3.3.2. Method II—Basic Catalyst

The reaction was conducted using an equimolar amount of thymol and formaldehyde (6.00 mmol) and 6 mL of NaOH (40%) for 2 h at 60 °C. After this period, the crude product was neutralized with acetic acid (1 mol·L^−1^), filtered, washed with H_2_O, and dried under reduced pressure at 70 °C to afford a brown material (Entry 5, Table 4). Additionally, the reaction was performed in the same way as mentioned above, but neutralized with HCl (1 mol·L^−1^) (Entry 6, Table 4). Furthermore, the reaction was carried out over 24 and 48 h (Entries 7 and 8, Table 4) and with temperatures of 40 and 80 °C (Entries 9 and 10, Table 4). All reactions provided yields of PTF from 19 to 62% (η). It is worth noting that the yields were calculated for the obtained PTF, regardless of the size of the polymer chains.

Poly(thymolformaldehyde) (PTF). ¹H NMR (300 MHz, CD_3_OD) δ_H_ 1.02–1.19 (m, H-9 and H-10), 2.10–2.31 (m, H-7), 3.15–3.33 (m, H-8), 3.72–4.16 (m, H-12), and 6.55–6.89 (m, H-2). ¹³C NMR (75 MHz, CD_3_OD) δ_C_ 17.9 (C-7), 22.2 (C-9 and C-10), 26.1 (C-8), 36.2 (C-12), 116.2 (C-5), 122.4 (C-1), 126.6 (C-2), 132.4 (C-3), 136.0 (C-6), and 151.2 (C-4). IR ν/cm^−1^: 4000–3390 (ν_C-H_ arom), 3225 (ν_O-H_ phenol), 3000–2870 (ν_C-H_ aliph), 1643 (ν_C=C_ arom), 1469 (δ_CH_2__), 1020 (ν_C-O_), and 952 (δ_CH_ oop).

### 3.4. Bioassay 1—Minimum Inhibitory Concentration (MIC)

A determination of the minimum inhibitory concentration (MIC) test was adopted by following a protocol from the literature [35]. All the samples were evaluated at concentrations ranging from 500 to 3.9 μg·mL^−1^. All the tested compounds were solubilized in ultrapure water and dimethyl sulfoxide (DMSO) (20%). Chloramphenicol^®^ (12 μg·mL^−1^) and Nystatin (200 I.U.) were used as reference commercial microbicides and applied as positive controls in the experiments. The microbicidal activity of all the compounds was evaluated against the following pathogens: *Escherichia coli* ATCC8739; *Staphylococcus aureus* ATCC 25923; *Pseudomonas aeruginosa* ATCC 9721; and *Candida albicans* ATCC40.006. All the analyses were performed in 96-well microplates, in triplicate, and as a form to ensure the accuracy and reproducibility of the results. This bioassay methodology can provide valuable information about the potency of the evaluated compounds against different pathogens and can help guide further research and the development of potential antimicrobial agents.

### 3.5. Bioassay 2—Cell Viability Assays and Biofilm Dispersion Tests

These tests were based on a previous study [36] with minor modifications. In our study, we aimed to quantify the biofilm architecture formed by the *Pseudomonas aeruginosa* (ATCC 1088) bacterium using two indirect quantitative methods: cell viability, measured using the fluorescent signal of resazurin metabolization into resorufin, and a colorimetric quantification of biomass, measured using crystal violet incorporation into the biofilm. The analyses were performed in microplates of 96 wells filled with 200 µL of an aqueous suspension of the bacterial model (*P. aeruginosa*) at a concentration of 1 × 10^8^ cells (OD_600nm_ = 0.08), pre-cultured in a brain–heart infusion (BHI, KASVI) culture medium. After 24 h of incubation at 37 °C and 150 rpm, the supernatant was removed, and 200 µL of culture medium was added to renew the nutrients. The same microplate was incubated again for another 24 h, at 37 °C and 150 rpm. After this period, the wells were washed with 1× PBS buffer.

Then, a solution of the bioactive compound, already solubilized in 10% dimethylsulfoxide (DMSO), was added to each well in a serial dilution, starting from the concentration of 500 µg·mL^−1^. The assay was again incubated for another 24 h under the same described conditions. A positive control of microbial growth and biofilm formation was performed by adding 1× PBS buffer, while a negative control (without microorganisms) was made using the BHI culture medium.

After the incubation period, cell viability was quantified by adding 20 μL of 200 μM resazurin to each well, followed by a new incubation at 37 °C for 25 min. All the results were obtained by readings performed in an ELISA-type spectrophotometer with a fluorescence filter at an excitation of λ 530 nm and an emission of λ 590 nm. Then, the resazurin supernatant was removed and the microplates were washed with 1× PBS buffer, followed by the fixation of the biofilm by adding methanol to each well, keeping it in contact with the biofilm for 15 min, and then pouring it off and drying the biofilm for 10 min.

Biomass quantification was performed by adding 150 µL per well of 0.1% crystal violet solution for 15 min. Then, the microplate was washed twice with distilled water and the final content was eluted with 95% ethanol for 30 min. The solution was transferred to a new 96-well microplate and absorbance readings were performed at a wavelength of 540 nm in a microplate reader. The results were calculated by the average of the readings obtained from the analyzed microwells, subtracting the values obtained for the experimental controls (wells containing only culture medium).

## 4. Conclusions

In this study, the polymerization of thymol was carried out using this monomer with formaldehyde in different proportions in several mediums (acid and basic). As a result, it was observed that the reaction performed using 40% NaOH afforded products with a higher purity and yield of PTF. Oligomers with similar characteristics such as optical and thermal properties, but with different chain sizes, were obtained. However, an improvement in a property of the macromolecules, the bioactivity against different pathogens, is highlighted. The synthesized macromolecules derived from thymol, which is widely used for bioapplications, presented significantly enhanced antimicrobial activity in comparison to the monomer. In this way, depending on the type of application for which it is necessary, the use of the macromolecule is suggested, since it brings great advantages compared to the use of thymol.

## Figures and Tables

**Table 1:** Reaction yield and obtained molar masses with polydispersity index (PDI) in each entry of the synthesis of PTF.

Entry	Yield/%	Molecular Mass (g·mol^−1^)/PDI
1	28	1295/1.75
2	83	650/1.38
3	51	793/1.56
4	62	673/1.46
5	38	488/1.87
6	62	1375/1.99
7	44	903/1.46
8	49	1106/1.59
9	19	373/1.40
10	34	715/1.44

**Table 2:** Events of thermal degradation of thymol, PTF/CHCl_3_, and PTF/CH_3_OH.

Events of Thermal Degradation			
Sample	Temperature Range/°C		Mass Loss/%
	Initial	Final	
Thymol	50	160	100
PTF/CHCl_3_	88	260	17
	260	530	76
PTF/CH_3_OH	30	80	6
	130	230	8
	230	330	37
	330	560	32

**Table 3:** MIC for thymol and PTF fractions tested against *Staphylococcus aureus*, *Escherichia coli*, *Pseudomonas aeruginosa*, and *Candida albicans* and molar masses (MM) with the polydispersity index (PDI) obtained using a GPC analysis to compare the data.

Sample	MIC (μg·mL^−1^)				GPC
	*S. aureus*	*E. coli*	*P. aeruginosa*	*C. albicans*	
Thymol	500	500	500	NA	MM = 150.22 g·mol^−1^
PTF/CHCl_3_	31.2	500	NA	NA	MM = 1182.0 g·mol^−1^ PDI = 1.79
PTF/CH_3_OH	62.5	500	500	500	MM = 1375.0 g·mol^−1^ PDI = 1.99

NA: non-active.

**Table 4:** Reaction parameters adopted to optimize the synthesis of PTF.

Entry	THY:FA *	Catalysis	Temperature/°C	Time/h	Treatment
1	1:1	Acid	60	4.00	NaOH
2	1:1	Acid	80 **	1.00	NaHCO_3_
3	1:1	Acid	80 **	3.00	NaHCO_3_
4	1:4	Acid	80 **	1.00	NaHCO_3_
5	1:1	Basic	60	2.00	CH_3_COOH
6	1:1	Basic	60	2.00	HCl
7	1:1	Basic	60	24.00	HCl
8	1:1	Basic	60	48.00	HCl
9	1:1	Basic	40	2.00	HCl
10	1:1	Basic	80	2.00	HCl

* thymol:formaldehyde; ** reaction conducted using microwave (MW).

## Data Availability

Data are contained within the article and Supplementary Materials.

## Supplementary Materials

The following supporting information can be downloaded at: https://www.mdpi.com/article/10.3390/molecules29051010/s1, Figure S1: LREIMS (70eV) data of thymol extracted from thyme essential oil; Figure S2: ¹H NMR spectrum of thymol (δ, CDCl_3_, 300 MHz); Figure S3: ¹³C NMR spectrum of thymol (δ, CDCl_3_, 75 MHz); Figure S4: Infrared spectra for thymol (black), PTF/CHCl_3_ (red) and PTF/CH_3_OH (blue); Figure S5: ¹H NMR spectrum of PTF (δ, CD_3_OH, 300 MHz); Figure S6: ¹³C NMR spectrum of PTF (δ, CD_3_OH, 75 MHz); Figure S7: TG curves for thymol (black), PTF/CHCl_3_ (red) and PTF/CH_3_OH (blue), ranging from 20 to 800 °C at 10 °C·min^−1^ and inert nitrogen atmosphere at 50 mL·min^−1^; Table S1: Fluorescent signal of the metabolism of resazurin to resofurin, comparing different concentrations of thymol, PTF/CHCl_3_, and PTF/CH_3_OH; Table S2: Colorimetric quantification of biomass, comparing different concentrations of thymol, PTF/CHCl_3_, and PTF/CH_3_OH.
