# Supplementary material for: Thymol as Starting Material for the Development of a Biobased Material with Enhanced Antimicrobial Activity: Synthesis, Characterization, and Potential Application
Source: Molecules. 2024 Feb 26;29(5):1010. doi: 10.3390/molecules29051010 (PMC10933892; doi:10.3390/molecules29051010)
Supplement: Supplementary file 1 [file molecules-29-01010-s001.zip › molecules-2666190-supplementary.pdf]

Thymol as starting material for the development of a biobased material with enhanced antimicrobial activity: synthesis, characterization, and potential application

Giovana A. Parolin[1], Vitor G. Vital[2], Suzan P. de Vasconcellos[2],

João Henrique G. Lago[3], Laura O. Péres[1*]

[1]*Laboratory of Hybrid Materials, Chemistry Department, Federal University of São Paulo, Diadema 09913-030, SP, Brazil,*

[2]*Multidisciplinary Laboratory of Health and Environment Sciences, Department of Pharmaceutical Sciences, Federal University of São Paulo, Diadema 09913-030, SP, Brazil,*

[3]*Laboratory of Chemical Biology, Center for Natural and Human Sciences, Federal University of ABC, Santo Andre 09210-580, SP, Brazil,*

Supporting Information

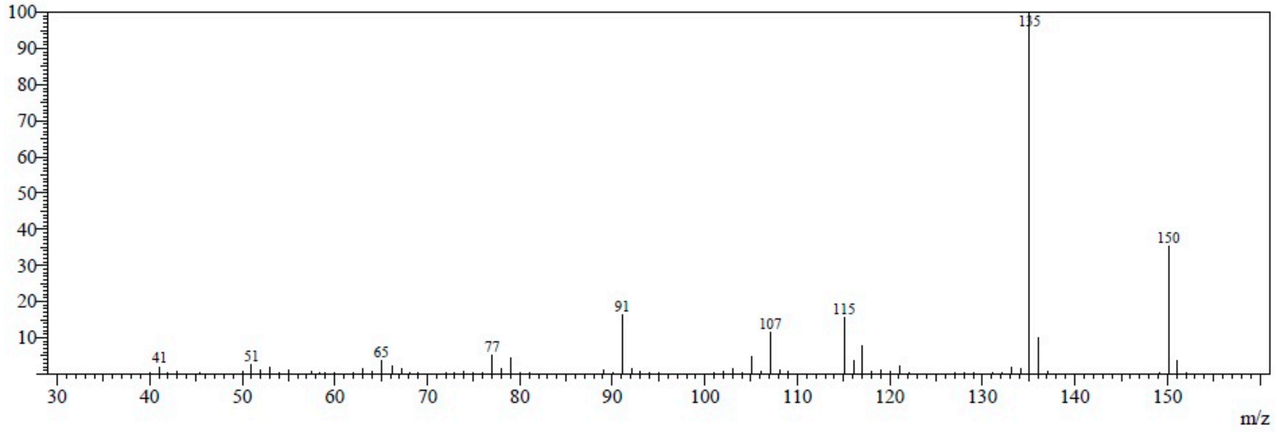

Figure S1. LREIMS (70eV) data of thymol extracted from thyme essential oil.

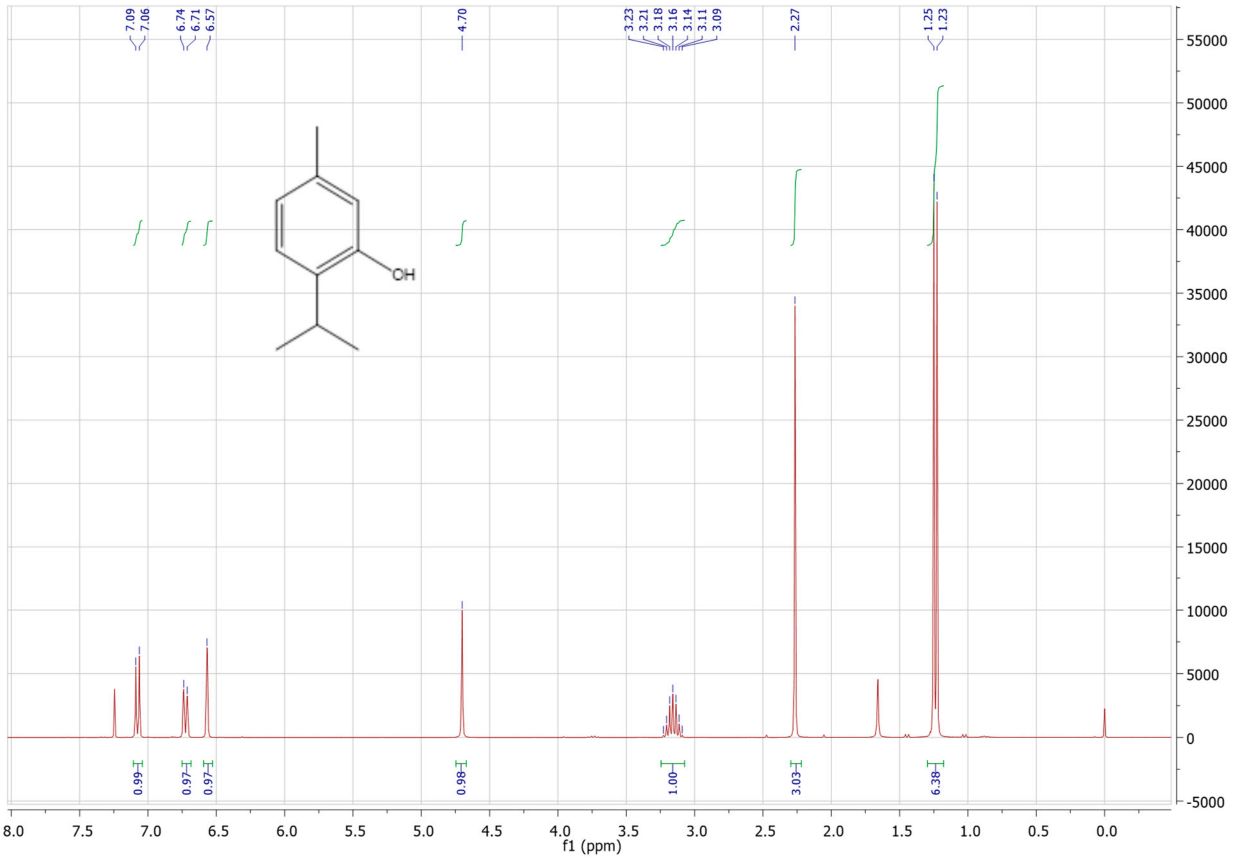

Figure S2. ^{1}H NMR spectrum of thymol (δ, $CDCl_3$, 300 MHz).

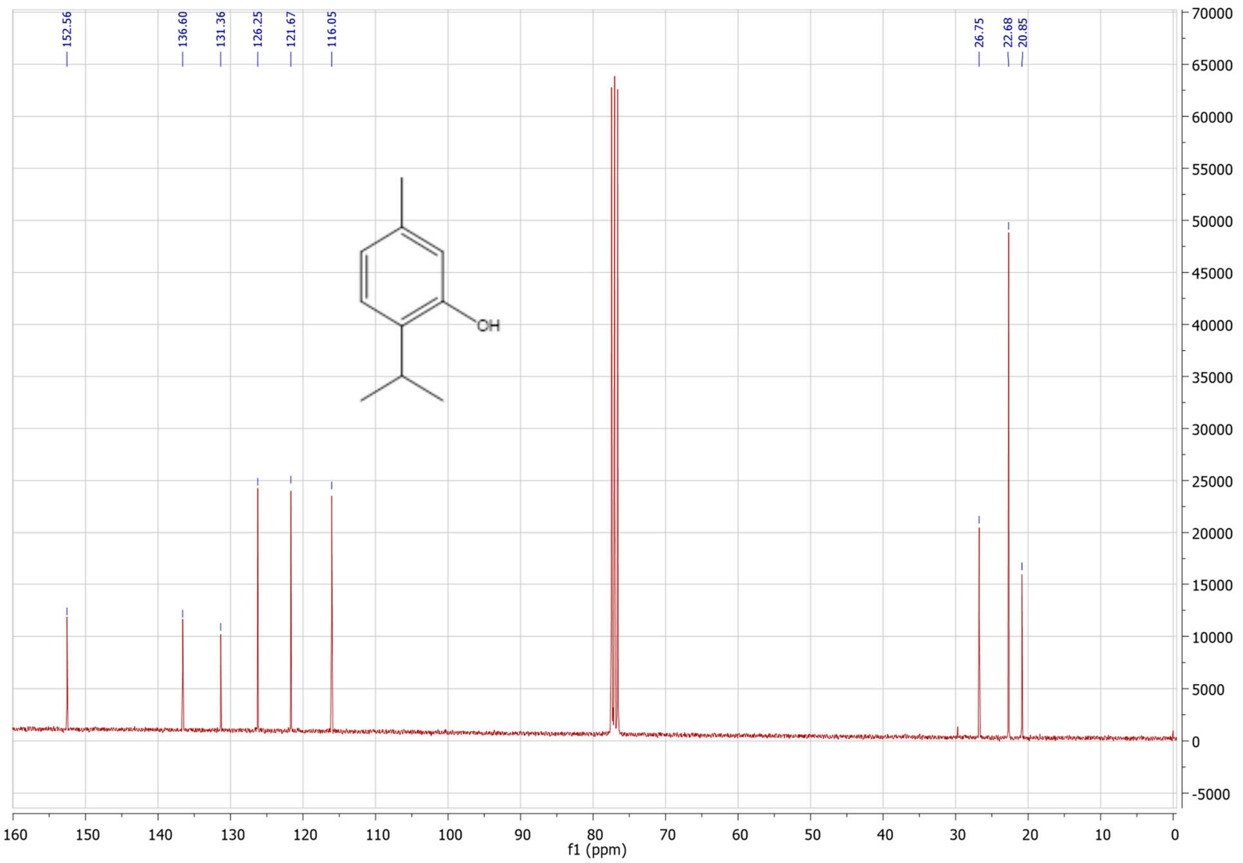

Figure S3. ^{13}C NMR spectrum of thymol (δ, $CDCl_3$, 75 MHz).

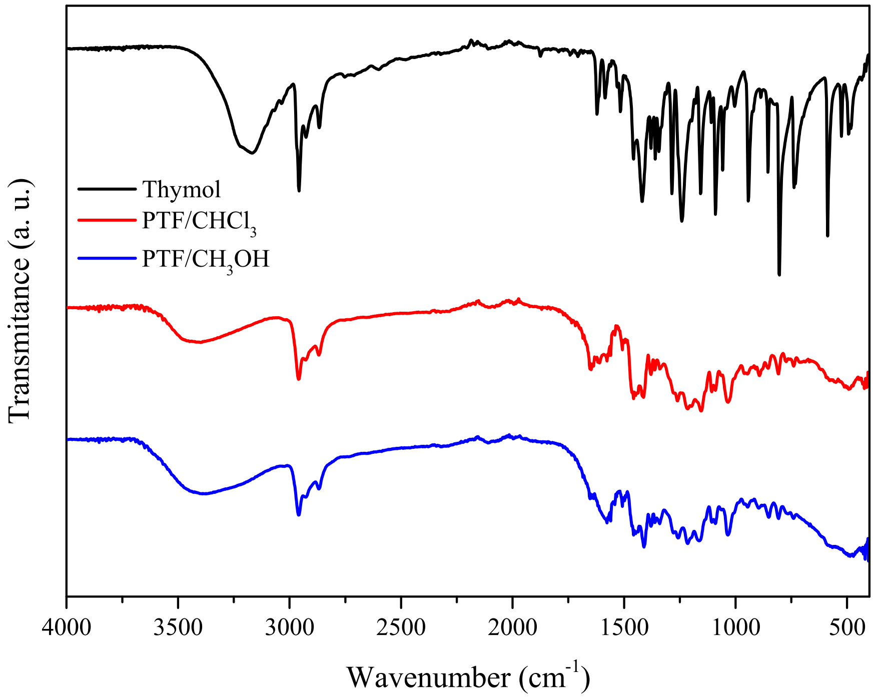

Figure S4. Infrared spectra for thymol (black), PTF/$CHCl_3$ (red) and PTF/CH_3OH (blue).

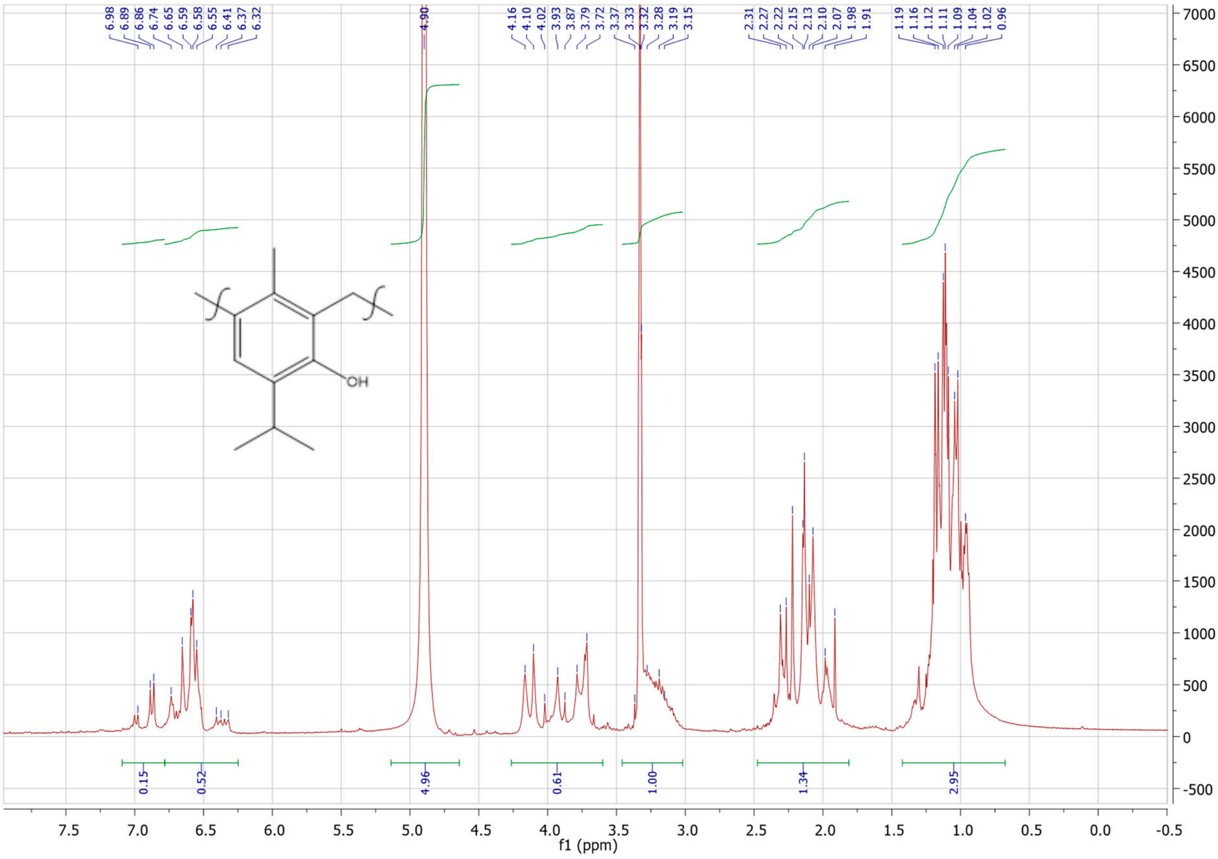

Figure S5. ^{1}H NMR spectrum of PTF (δ, CD_3OD, 300 MHz).

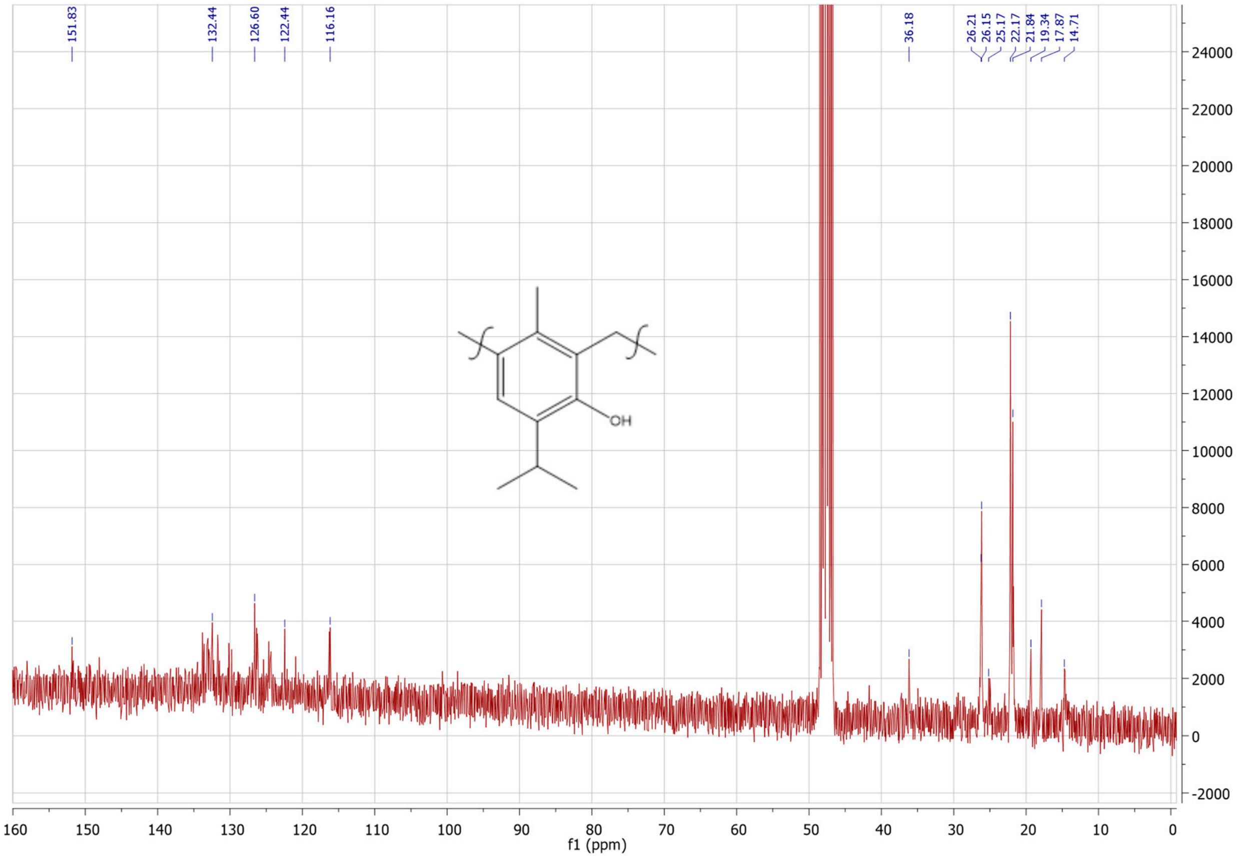

Figure S6. ^{13}C NMR spectrum of PTF (δ, CD_3OD, 75 MHz).

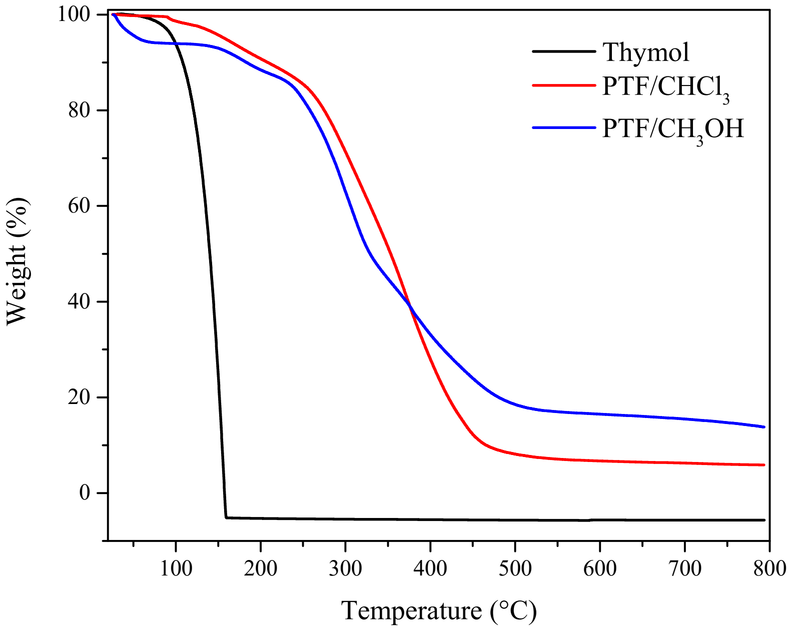

Figure S7. TG curves for thymol (black), PTF/$CHCl_3$ (red) and PTF/CH_3OH (blue), ranging from 20 to 800 °C at 10 °C.min^{-1} and inert nitrogen atmosphere at 50 mL.min^{-1}.

Table S1: Fluorescent signal of the metabolism of resazurin to resofurin, comparing different concentrations of thymol, PTF/$CHCl_3$, and PTF/CH_3OH.

$\mu g.mL^{-1}$	*Thymol*	*PTF/$CHCl_3$*	*PTF/CH_3OH*	*Control*
**500.00**	507 ± 176	590 ± 190	338 ± 100	1542 ± 378
**250.00**	1483 ± 252	1264 ± 185	922 ± 422	
**125.00**	1734 ± 299	1370 ± 166	1377 ± 258	
**62.50**	1945 ± 371	1699 ± 140	1410 ± 190	
**31.25**	1795 ± 539	1607 ± 229	1418 ± 250	
**15.63**	2354 ± 436	2017 ± 90	2011 ± 47	
**7.81**	2035 ± 289	1897 ± 93	1820 ± 86	
**3.91**	2639 ± 356	2540 ± 117	2918 ± 329	

Table S2: Colorimetric quantification of biomass, comparing different concentrations of thymol, PTF/$CHCl_3$, and PTF/CH_3OH.

$\mu g.mL^{-1}$	*Thymol*	*PTF/$CHCl_3$*	*PTF/CH_3OH*	*Control*
**500.00**	0.14 ± 0.02	0.15 ± 0.02	0.18 ± 0.06	0.16 ± 0.05
**250.00**	0.15 ± 0.03	0.20 ± 0.01	0.15 ± 0.02	
**125.00**	0.15 ± 0.02	0.16 ± 0.02	0.14 ± 0.01	
**62.50**	0.13 ± 0.02	0.13 ± 0.01	0.20 ± 0.01	
**31.25**	0.11 ± 0.02	0.11 ± 0.01	0.15 ± 0.01	
**15.63**	0.10 ± 0.02	0.12 ± 0.03	0.12 ± 0.04	
**7.81**	0.12 ± 0.03	0.12 ± 0.01	0.16 ± 0.00	
**3.91**	0.14 ± 0.04	0.15 ± 0.03	0.18 ± 0.00	
